# COVID-19, tuberculosis, and HIV triad: a prospective observational study in ambulatory patients in Kenya, Uganda, and South Africa

**DOI:** 10.1371/journal.pgph.0004471

**Published:** 2025-04-23

**Authors:** Helena Huerga, Maelenn Gouillou, Liesbet Ohler, Ivan M. Taremwa, Milcah Akinyi, Alex Lubega, Winnie R. Muyindike, Mathieu Bastard, Claire Bossard, May Atieno, Rose Muhindo, Esther C. Casas, Lydia Nakiyingi, Martina Casenghi, Ankur Gupta-Wright, Maryline Bonnet, Zibusiso Ndlovu

**Affiliations:** 1 Epicentre, Paris, France,; 2 Médecins Sans Frontières, Eshowe, South Africa,; 3 Epicentre, Mbarara, Uganda,; 4 Médecins Sans Frontières, Homa Bay, Kenya,; 5 Mbarara Regional Referral Hospital, Mbarara University of Science and Technology, Mbarara, Uganda,; 6 Mbarara University of Science and Technology, Mbarara, Uganda,; 7 Médecins Sans Frontières, Southern Africa Medical Unit, Cape Town, South Africa,; 8 Makerere University College of Health Sciences, Kampala, Uganda,; 9 Elizabeth Glaser Pediatric AIDS Foundation, Geneva, Switzerland,; 10 Imperial College, North Bristol NHS Trust, London, UK,; 11 Institut de Recherche pour le Développement, University of Montpellier, Montpellier, France,; 12 Médecins Sans Frontières, Southern Africa Medical Unit, Cape Town, South Africa; Division of Epidemiology and Biostatistics, Department of Global Health, Faculty of Medicine and Health Sciences, Stellenbosch University, Cape Town, South Africa; New York University Grossman School of Medicine, UNITED STATES OF AMERICA

## Abstract

People living with HIV (PLHIV) have an increased risk of tuberculosis (TB) and severe COVID-19. TB and COVID-19 present with overlapping symptoms and co-infection can lead to poor outcomes. We assessed the frequency of SARS-CoV-2 positive serology and SARS-CoV-2 infection and the risk of mortality at 6 months in PLHIV with TB disease and SARS-CoV-2 infection. This multi-country, prospective, observational study, conducted between 7^th^ September 2020 and 7^th^ April 2022, included ambulatory adult PLHIV investigated for TB (with symptoms of TB or advanced HIV disease) in Kenya, Uganda, and South Africa. Testing included CD4 cell count, Xpert MTB/RIF Ultra assay (sputum), Determine TB LAM Ag assay (urine), chest X-ray, blood SARS-CoV-2 serology test and SARS-CoV-2 PCR (only if TB or COVID-19 symptoms). Individuals were followed for 6 months. Among 1254 participants, 1204 participants had SARS-CoV-2 serology (54% women, median CD4 344 cells/µL [IQR 132–673]), and 487 had SARS-CoV-2 PCR. SARS-CoV-2 serology positivity was 27.0% (325/1204), lower in PLHIV with CD4 counts <200 cells/µL (19.9%, 99/497) than in those with CD4 counts ≥200 cells/µL (31.6%, 222/703), p<0.001. SARS-CoV-2 PCR positivity was 8.6% (42/487) and 27.7% (135/487) had probable or confirmed SARS-CoV-2 infection. Among PLHIV with symptoms of TB or of COVID-19, 6.6% (32/487) had SARS-CoV-2 infection and TB disease. In multivariable analyses, the risk of death was higher in PLHIV with both SARS-CoV-2 infection and TB compared to those with only SARS-CoV-2 infection (adjusted hazard ratio [aHR] 8.90, 95%CI 1.47-53.96, p=0.017), with only TB (aHR 3.70, 95%CI 1.00-13.72, p=0.050) or with none of them (aHR 6.83, 95%CI 1.75-26.72, p=0.006). These findings support SARS-CoV-2 testing in PLHIV with symptoms of TB, and SARS-CoV-2 vaccination, especially for those with severe immunosuppression. PLHIV with COVID-19 and TB have an increased risk of mortality and would benefit from comprehensive management and close monitoring.

## Background

More than half of the 39.9 million people living with HIV (PLHIV) and 25% of new global TB cases are in Africa [1]. Collectively, HIV/AIDS and TB are the deadliest infectious diseases. Since its emergence in 2019 until 2023, SARS-CoV-2 has infected over 12 million people in Africa with over 250,000 deaths [2]. The COVID-19 pandemic devastated health systems and had a huge knock-on effect on HIV, TB, malaria and other communicable and non-communicable diseases [3]. The combined challenges posed by COVID-19, TB, and HIV represent a significant and ongoing global health crisis.

COVID-19 can have unique implications for certain populations and PLHIV have an increased risk of severe or critical COVID-19 disease and mortality compared with HIV-negative individuals [4,5]. Vulnerability to COVID-19 and immune-response to the disease can differ according to the level of immunosuppression in PLHIV [6]. In addition, TB and COVID-19 can both present with lung involvement and overlapping symptoms which may make differential diagnosis difficult [7]. Several studies have shown that co-infection with TB and COVID-19 lead to poorer outcomes than each of them alone [5,8–13]. In 2024, SARS-CoV-2 vaccine coverage rates in Africa were around 50% [14]. Some SARS-CoV-2 strains are associated with substantial ability to evade immunity from prior infection and vaccines [15]. These findings underscore that SARS-CoV-2 has propensity to resurge in the future. Also, up to 30% of the people infected by SARS-CoV-2 may go on to develop symptoms that can be diagnosed as long COVID-19 [16,17] Consequently, there is a need for further research to enhance understanding about COVID-19, especially in the context of individuals living with HIV and or TB to support future public health planning. This study assessed the frequencies of SARS-CoV-2 positive serology and SARS-CoV-2 infection, and the variation by CD4 cell count, in PLHIV investigated for TB in Kenya, Uganda, and South Africa. In addition, mortality at 6 months in PLHIV with/without TB or COVID-19 was investigated.

## Methods

### Study design and population

This multi-country, prospective, observational study was conducted between 7^th^ September 2020 and 7^th^ April 2022. The study consecutively included ambulatory adult PLHIV (aged ≥15 years) investigated for TB, with either signs or symptoms of TB or no symptoms of TB but advanced HIV disease (CD4 count <200 cells/μL, or WHO HIV clinical stage 3 or 4), who provided written informed consent (or assent for minors aged 15–17 years), and had SARS-CoV-2 testing with either serology, PCR, or both. Signs and symptoms of TB were cough, fever, weight loss, night sweats, or symptoms of extrapulmonary TB of any duration. Patients previously diagnosed with TB and receiving TB treatment were excluded. The study was nested into a multi-country study of TB diagnostics among PLHIV [18].

### Study sites

Participants were recruited in four sites in Kenya, Uganda, and South Africa. In Kenya, participants were recruited at the HIV and TB clinics attached to Homa Bay County Hospital in Western Kenya, a referral facility managed by the Kenyan Ministry of Health. Homa Bay subcounty has an estimated population of 117,000 people [19]. In Uganda, participants were recruited at the HIV clinic attached to Mbarara Regional Referral Hospital in Western Uganda, a public and teaching hospital managed by the Ugandan Ministry of Health. Mbarara District has an estimated population of 472,000 people [20]. In South Africa, participants were recruited at the HIV and TB clinic attached to Eshowe District Referral Hospital and at the Eshowe Gateway Clinic, a Primary Health Care Clinic in Eshowe, uMlalazi, KwaZulu-Natal. Both are managed by the South African Department of Health. uMlalazi municipality has around 215,000 people [21]. The first SARS-CoV-2 vaccines were available in South Africa, Kenya and Uganda between February and March 2021 [22]. Vaccination was offered to the general population though in some countries health workers and vulnerable populations were initially targeted or prioritized [23].

### Study procedures

Eligible individuals were asked for written informed consent prior to inclusion in the study. At the initial visit, all patients received a clinical examination with particular focus on SARS-CoV-2, TB-related signs and symptoms, and signs of severity. SARS-CoV-2 vaccine information (type of vaccine and vaccination date) was self-reported or collected using vaccine cards. Participants had a CD4 count performed using Pima Analyser (Abbott, USA) or FacsCalibur (Becton Dickinson, USA). All participants were offered SARS-CoV-2 serological testing of venous blood samples using SARS-CoV-2 Abbot IgG ELISA assay (the test used did not differentiate between vaccination and past infection). SARS-CoV-2 PCR testing in a nasopharyngeal sample using conventional (ABI Quant Studio 5 PCR and ABI 7500 Fast Real-Time PCR) or point-of-care (GeneXpert SARS-CoV-2) PCR was offered to all participants with symptoms of TB and to patients without symptoms of TB but with COVID-19 related symptoms (runny nose, sore throat, loss of smell, loss of taste, headache, fatigue, difficulty to breath). All individuals (whether with symptoms of TB or asymptomatic) were investigated for TB through systematic Xpert MTB/RIF Ultra assay (Xpert Ultra; Cepheid, Sunnyvale, USA) on sputum, Determine TB LAM Ag assay (AlereTB-LAM; Abbott, USA) on urine, and chest X-ray. Xpert Ultra (or culture in South Africa) was performed on urine for those unable to produce sputum, and on other non-respiratory samples for patients with signs of extrapulmonary TB. Additionally, in Uganda, sputum was systematically cultured using the Mycobacterial Growth Indicator Tube (MGIT) liquid culture (Becton Dickinson, USA) and Lowenstein-Jensen medium solid culture. In South Africa, MGIT liquid culture was used.

All participants were followed up for 6 months. Individuals who had not started TB treatment at the initial visit had a follow-up consultation at 1 week. At this visit, they had a clinical exam and repeated TB investigations if needed. All participants had follow-up visits at 3 and 6 months, which included a clinical examination. At the 6-months visit, all participants were systematically offered SARS-CoV-2 serological testing.

Individuals diagnosed with SARS-CoV-2 infection were referred to the local Ministry of Health COVID-19 facilities for management. The Ministry of Health COVID-19 treatment units in the study sites were responsible for patient care and provided COVID-19 care following the national guidelines in each country.

### Definitions

Patients diagnosed with TB included those with probable or confirmed TB. Confirmed TB was defined as having a positive Xpert MTB/RIF or MTB culture result in any body sample either at the initial consultation or during follow-up. Probable TB encompassed patients who did not meet the criteria for confirmed TB but were initiated on TB treatment either at the initial consultation or during follow-up based on clinical or radiological findings.

Patients diagnosed with SARS-CoV-2 infection included those with probable or confirmed SARS-CoV-2 infection. Confirmed SARS-CoV-2 infection was defined as having a positive Nucleic Acid Amplification Test (positive SARS-CoV-2 PCR). As per WHO definition [24], probable SARS-CoV-2 infection included individuals with acute onset of fever and cough or acute onset of any three or more of the following signs or symptoms: fever, cough, general weakness/fatigue, headache, myalgia, sore throat, coryza, dyspnoea, nausea/diarrhoea/anorexia (signs separated with slash were counted as one sign) or patients with severe acute respiratory illness. Acute onset was defined as onset within the last 10 days.

Positive SARS-CoV-2 IgG serology test was defined as a positive SARS-CoV-2 Abbott serological test at the initial consultation or during follow-up.

Patients seriously ill were those with temperature of >39°C, a respiratory rate of >30 respirations per min, a cardiac rate of >120 beats per minute, or inability to walk without help.

### Outcomes

The primary outcomes of the study were the frequencies of positive SARS-CoV-2 serological testing and of SARS-CoV-2 infection (confirmed and probable) throughout the study period (outcome 1). Secondary outcomes were the frequency of positive SARS-CoV-2 serological testing over time (during the study period), and the frequency of positive SARS-CoV-2 serological testing and of SARS-CoV-2 infection by CD4 count level (<200 and ≥200 cells/mm^3^) and by TB diagnosis (confirmed and probable) (outcome 2). Lastly, the study sought to determine the risk of all-cause mortality over 6 months among PLHIV diagnosed with either TB, SARS-CoV-2 infection, or both, compared to PLHIV with neither co-infection in a subset of individuals with symptoms of TB or of COVID-19 (outcome 3).

### Sample size

To estimate a frequency of positive SARS-CoV-2 serology among PLHIV of 3% with a precision of 1%, an alpha error of 0.05 and a power of 80%, at least 1120 individuals were needed. To estimate a frequency of SARS-CoV-2 infection among PLHIV with symptoms of TB or of COVID-19, of 10% with a precision of 3%, an alpha error of 0.05 and a power of 80% using a Wilson score formula with continuity correction, at least 420 individuals were needed.

### Statistical analyses

Study participants with at least one test result for SARS-CoV-2 serology or PCR were included in the analyses. Individuals’ characteristics for participants with SARS-CoV-2 serology results and for those with SARS-CoV-2 PCR results were described as presented at the initial consultation. Continuous variables were summarized as median and interquartile range (IQR) and categorical variables as absolute numbers and percentages. Positivity of SARS-CoV-2 serological testing, positivity of SARS-CoV-2 PCR and SARS-CoV-2 infection were calculated overall, and according to CD4 count level and to TB diagnosis. As positivity of SARS-CoV-2 serological testing varied over time, this indicator was also calculated per month and per periods of 4 months: September to December 2020, January to April 2021, May to August 2021, September to December 2021, and January to April 2022. Chi-Square or Fisher’s exact tests, as appropriate, were used to compare independent proportions.

Participants with signs and symptoms of TB or of COVID-19 were included in the mortality analyses. PLHIV were classified as having only SARS-CoV-2 infection, only TB disease, or both SARS-CoV-2 infection and TB. Crude mortality rates were calculated in each group. Mortality over the 6-month follow-up was described using Kaplan-Meier curves and log-rank tests. For all patients, person time was accrued from date of enrollment into the study until the earliest of mortality, loss to follow-up (LTFU), or end of follow-up study period. Patients LTFU were censored at their last health facility visit date. In addition, a Cox proportional hazards model was used to explore the risk of 6-month mortality for PLHIV with SARS-CoV-2 infection, TB and both SARS-CoV-2 and TB, using ‘only HIV infected’ as reference. Causal diagrams were used to identify potential confounders. Age, sex, BMI, HIV viral load, CD4 cell count, diabetes, and country, were identified as potential confounders (S1 Fig). When two variables showed collinearity, only the most appropriate in relation with mortality and considering also completeness and reliability was selected. The model included age, sex, BMI, CD4 cell count, and country. Missing data were included in the model using specific categories. Adjusted hazard ratios and 95% confidence intervals of mortality were estimated. Proportional hazards (PH) assumption was checked by testing the Schoenfeld residuals, and model goodness of fit was tested using Cox-Snell residual. P-values <0.05 were considered statistically significant.

Analyses were performed using Stata 18 software (Stata Corporation, College Station, Texas, USA).

### Ethical considerations

The study protocol was approved by the local Ethics Committees in Kenya, Uganda and South Africa as well as by Médecins Sans Frontières Ethics Review Board. Written informed consent (or assent for minors aged 15–17 years) was obtained from all study adult participants and from parents or guardians for minors.

### Inclusivity in global research

Additional information regarding the ethical, cultural, and scientific considerations specific to inclusivity in global research is included in the Supporting Information (S1 Text).

## Results

### Participants’ characteristics

**Fig 1:**
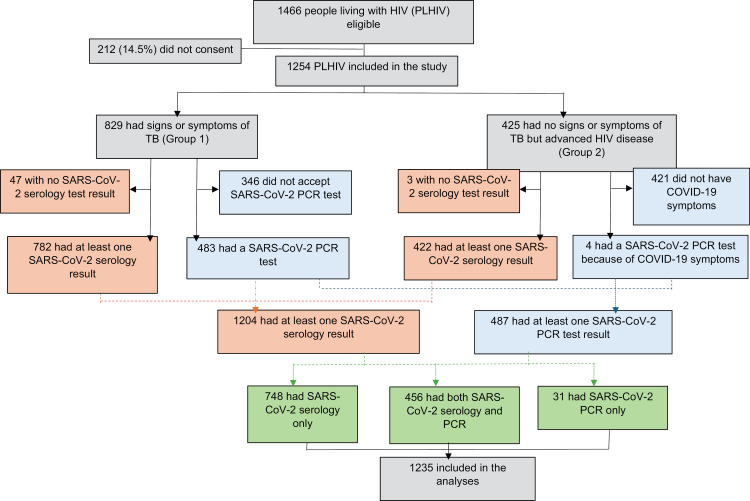
Patients’ flow.

In total, 1254 (85.5%) of 1466 PLHIV investigated for TB provided consent and were included in the study (Fig 1). All participants were eligible for SARS-CoV-2 serology and were tested. Of them, 1204 (97.5%) had a result. Of the 829 participants eligible for SARS-CoV-2 PCR testing (individuals with symptoms of TB), 483 (58%) accepted the test and all had a result. In addition, 4 individuals without symptoms of TB but with COVID-19 symptoms were also tested. The clinical-demographical characteristics of the individuals who accepted and refused SARS-CoV-2 PCR testing were similar, except for being seriously ill (among refusals, lower proportion) and country (among refusals, higher proportion of participants from Kenya and lower from South Africa), S1 Table. Overall, 1235 individuals were included in the analyses, 1204 had at least one SARS-CoV-2 serology result and 487 had a SARS-CoV-2 PCR result.

The characteristics of the participants are presented in Table 1. Among the 1204 patients with serology results, 645 (54%) were female. Median age was 41 years (IQR 33–50) and median CD4 was 344 cells/µL (IQR 132–673). Few patients were vaccinated against SARS-CoV-2, 32 (2.7%) at the first visit, and 83 (6.9%) at any point during follow-up. Half of them (56%) were vaccinated between November 2021 and February 2022. Among the 487 patients with SARS-CoV-2 PCR results, 299 (61%) were women. Median CD4 was 555 cells/µL (IQR 313–784).

**Table 1:** Participants’ characteristics at the first visit for people living with HIV tested with SARS-CoV-2 serology and with SARS-CoV-2 PCR.

	Tested with SARS-CoV-2 serology^*^ (N=1204)		Tested with SARS-CoV-2 PCR^*^ (N=487)	
	n	% or IQR^†^	n	% or IQR^†^
**Demographics**				
Women	645	53.6%	299	61.4%
Men	559	46.4%	188	38.6%
Age, median [IQR]	41	33–50	42	34–52
**CD4 count**				
Median, cells/µL [IQR]	344	132–673	555	313–784
<200 cells/µL	497	41.3%	81	16.6%
≥200 cells/µL	703	58.4%	405	83.2%
Missing	4	0.3%	1	0.2%
**Viral load**				
≤1000 copies/mL	630	52.3%	327	67.1%
>1000 copies/mL	107	8.9%	21	4.3%
Missing	467	38.8%	139	28.5%
**Antiretroviral therapy initiated**				
Yes	1092	90.7%	442	90.8%
No	106	8.8%	39	8.0%
Missing	6	0.5%	6	1.2%
**Reason for tuberculosis (TB) investigation**				
Symptoms of TB	782	65.0%	483	99.2%
No symptoms of TB but advanced HIV disease	422	35.0%	4	0.8%
**Comorbidities**				
Cardiovascular disease	3	0.3%	2	0.4%
Hypertension	32	2.7%	26	5.3%
Diabetes	14	1.2%	12	2.5%
Chronic lung disease	4	0.3%	3	0.6%
Asthma	12	1.0%	6	1.2%
Current smoker	70	5.8%	43	8.8%
Liver chronic disease	2	0.2%	1	0.2%
**Body-Mass Index**				
<17 kg/m²	121	10.1%	42	8.6%
17–18.4 kg/m²	123	10.2%	37	7.6%
18.5–24.9 kg/m²	662	55.0%	259	53.2%
25.0–29.9 kg/m²	186	15.5%	74	15.2%
≥30 kg/m²	106	8.8%	68	14.0%
Missing	6	0.5%	7	1.4%
**Clinical examination**				
Temperature ≥38°C	24	2.0%	16	3.3%
Heart rate >100 beats per min	168	14.0%	85	17.5%
Respiratory rate >20 breaths per min	396	32.9%	148	30.4%
Systolic blood pressure <90 mmHg	23	1.9%	11	2.3%
Seriously ill	42	3.5%	34	7.0%
**Vaccinated against SARS-COV-2**				
Yes	32	2.7%	9	1.9%
No	925	76.8%	435	89.3%
Missing	247	20.5%	43	8.8%
**Country**				
Uganda	686	57.0%	273	56.1%
Kenya	379	31.5%	79	16.2%
South Africa	139	11.5%	135	27.7%

* 456 patients had both SARS-CoV-2 serology and PCR, 748 only serology and 31 only PCR (N=1235 total patients).

† Interquartile Range (IQR).

### Proportion of SARS-CoV-2 positive serology and SARS-CoV-2 infection

**Fig 2:**
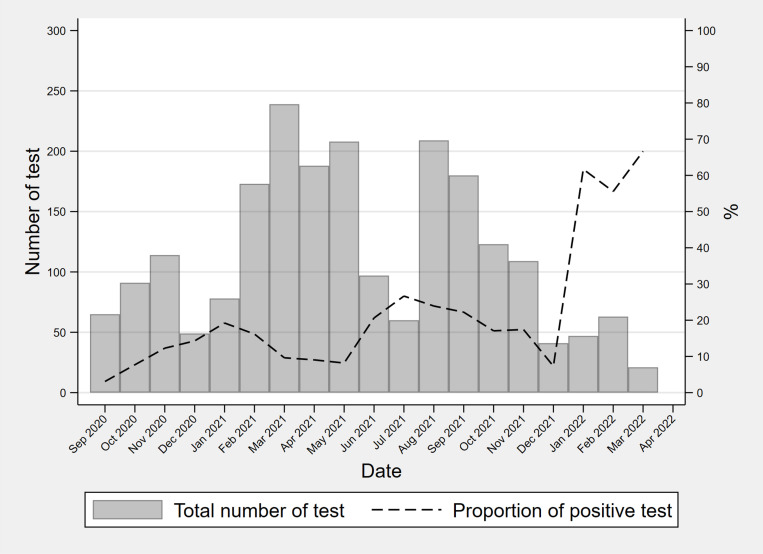
SARS-CoV-2 serological tests performed and positivity rate from September 2020 to April 2022 in people living with HIV investigated for TB.

Overall, 27.0% (325/1204) of participants had at least one positive SARS-CoV-2 serology result. The SARS-CoV-2 serology positivity rate increased over time and was 56.6% in Uganda, 40.0% in Kenya, and 71.1% in South Africa, by the last study period (January–April 2022) (Fig 2 and S2 Table). Of the individuals with initial positive serology, 44.4% (52/117) remained positive at 6-month testing. Among individuals with initial negative serology, 20.3% (168/826) had a positive test at 6-month testing. Positivity at 6-month among initially negative was 43.3% (26/60) among vaccinated and 18.6% (142/763) among non-vaccinated (p<0.001).

Among participants with symptoms of TB or COVID-19, tested with SARS-CoV-2 PCR, 8.6% (42/487) were positive. Among patients with symptoms of TB, 8.3% (40/483) had a positive SARS-CoV-2 PCR, 8.9% (24/269) in Uganda, 7.6% (6/79) in Kenya, and 7.4% (10/135) in South Africa, and among the four patients without symptoms of TB but with COVID-19-related symptoms, two were positive. In addition, 445 individuals with negative PCR, 93 (20.9%) met the WHO definition of probable SARS-CoV-2 infection. In total, 135/487 (27.7%) had SARS-CoV-2 infection (probable or confirmed).

### SARS-CoV-2 positive serology and SARS-CoV-2 infection by CD4 count

SARS-CoV-2 serology positivity rates were lower in patients with CD4 counts <200 cells/µL (19.9%, [99/497]) than in those with CD4 counts ≥200 cells/µL (31.6% [222/703]; p<0.001; Table 2). Among patients with an initial negative serological test, patients with CD4 counts <200 cells/µL had lower serological positivity rates at 6 months than those with CD4 counts ≥200 (16.4% [59/359] vs 23.0% [107/465], p=0.020). Among those with a positive serology test at baseline, serological positivity rates at 6 months by CD4 count level were 41.9% (13/31) for PLHIV with CD4 counts <200 cells/µL and 45.3% (39/86) for those with CD4 counts ≥200 cells/µL (p=0.743).

**Table 2:** SARS-CoV-2 serology and SARS-CoV-2 PCR positivity and SARS-CoV-2 infection by CD4 count in people living with HIV.

	All^*^		CD4 <200 cells/µL		CD4≥200 cells/µL		p-value
	n/N	% [95% CI]	n/N	% [95% CI]	n/N	% [95% CI]	
Positive SARS-CoV-2 serology	325/1204	27.0% [24.5; 29.5]	99/497	19.9% [16.4; 23.4]	222/703	31.6% [28.1; 35.0]	<0.001
Positive SARS-CoV-2 serology 6 months after negative result	168/826	20.3% [17.6; 23.1]	59/359	16.4% [12.6; 20.3]	107/465	23.0% [19.2; 26.8]	0.020
Positive SARS-CoV-2 serology 6 months after positive result	52/117	44.4% [35.4; 53.4]	13/31	41.9% [24.6; 59.3]	39/86	45.3% [34.8; 55.9]	0.743
Positive SARS-CoV-2 PCR^†^	40/483	8.3% [5.8; 10.7]	8/77	10.4% [3.6; 17.2]	32/405	7.9% [5.3; 10.5]	0.468
SARS-CoV-2 infection (probable or confirmed) ^‡^	135/487	27.7% [23.7; 31.7]	23/81	28.4% [18.6; 38.2]	112/405	27.7% [23.3; 32.0]	0.892

* In four patients CD4 was not available.

† Among patients with symptoms of TB.

‡ Among patients with symptoms of TB or COVID-19 related symptoms.

SARS-CoV-2 PCR positivity rates were 10.4% (8/77) in PLHIV with CD4 counts <200 cells/µL and 7.9% (32/405) in those with CD4 counts ≥200 cells/µL (p=0.468). SARS-CoV-2 infection rates were similar between patients with CD4 counts <200 cells/µL (28.4% [23/81]) and those with CD4 counts ≥200 cells/µL (27.7% [112/405]; p=0.892.

Vaccination rates by CD4 count level were: 5.9% (29/490) in patients with CD4 <200 cells/µL vs 7.7% (54/701) in those with CD4 counts ≥200 cells/µL (p=0.234).

### SARS-CoV-2 infection and TB disease among individuals with symptoms of TB or of COVID-19

Among the subset of 487 individuals with symptoms of TB or symptoms of COVID-19, 27.7% (135/487) were diagnosed with SARS-CoV-2 infection and 23.8% (116/487) were diagnosed with TB. The rates of SARS-CoV-2 infection were similar between patients diagnosed with TB (27.6% 32/116) and those without TB (27.8%, 103/371), p=0.970 (Table 3). SARS-CoV-2 PCR positivity was 12.9% (15/116) among patients diagnosed with TB and 7.3% (27/371) among those without TB (p=0.058).

**Table 3:** SARS-CoV-2 positive PCR and SARS-CoV-2 infection by tuberculosis (TB) diagnosis in the subset of individuals living with HIV presenting with symptoms of TB or of COVID-19.

	Not TB		TB^*^		Confirmed TB		Probable TB		p-value^†^
	n	% [95% CI]	n	% [95% CI]	n	% [95% CI]	n	% [95% CI]	
Positive SARS-CoV-2 PCR	27/371	7.3% [4.6; 9.9]	15/116	12.9% [6.8; 19.0]	5/39	12.8% [2.3; 23.3]	10/77	13.0% [5.5; 20.5]	0.058
SARS-CoV-2 infection (probable or confirmed)	103/371	27.8% [23.2; 32.3]	32/116	27.6% [19.4; 35.7]	9/39	23.1% [9.9; 36.3]	23/77	29.9% [19.6; 40.1]	0.970

* TB diagnosis includes probable and confirmed TB.

† P-value comparing proportions of Not TB and TB.

**Fig 3:**
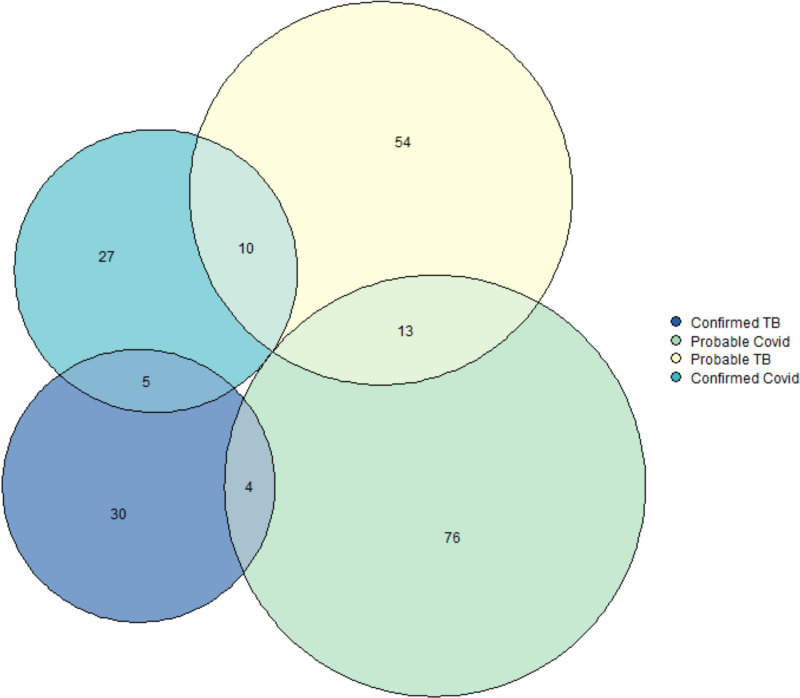
Euler diagram of people living with HIV presenting with symptoms of TB or of COVID-19 and with tuberculosis or SARS-CoV-2 infection.

In total, 55.0% (268/487) had only HIV, 21.1% (103/487) had HIV and SARS-CoV-2 infection, 17.2% (84/487) had HIV and TB disease, and 6.6% (32/487) had HIV and both SARS-CoV-2 infection and TB (Fig 3).

### Mortality at 6 months according to SARS-CoV-2 infection and TB disease among individuals with symptoms of TB or of COVID-19

Mortality over the 6-month follow-up period was 4.1% (n=20) in the subset of 487 participants with symptoms of TB or of COVID-19 investigated for SARS-CoV-2 infection and TB disease. Median follow-up was 5.6 months (IQR: 5.5-6.4). Twelve patients were lost-to-follow-up after the initial visit. A description of the deceased patients is shown in the Supporting information file, S3 Table).

**Fig 4:**
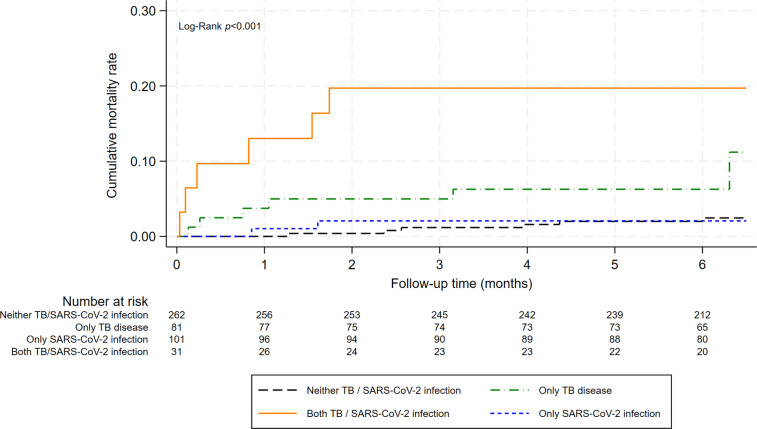
Mortality over 6 months according to SARS-CoV-2 infection and TB disease in people living with HIV presenting with symptoms of TB or of COVID-19 (log-rank test p<0.001).

Mortality was higher in PLHIV with both SARS-CoV-2 infection and TB (18.8%, 6/32) than in those with only SARS-CoV-2 infection (2.0%, 2/103, p<0.001), those with only HIV (2.2%, 6/268, p<0.001) and with only TB (7.1%, 6/84, p=0.067) (Fig 4). Median time to death from the first visit was 0.5 months (IQR 0.1-1.5) in PLHIV with both SARS-CoV-2 infection and TB, 1.2 months (IQR 0.9-1.6) in those with HIV and SARS-CoV-2 infection, 0.9 months in those with HIV and TB (IQR 0.3-3.2) and 3.2 (IQR 2.4-4.4) in those with only HIV.

In multivariable analyses, PLHIV with symptoms of TB or of COVID-19, and both SARS-CoV-2 infection and TB had an increased risk of death over 6 months compared with those with only HIV (adjusted hazard ratio [aHR] 6.83, 95% CI 1.75-26.72, p=0.006; Table 4). In separate multivariable models, PLHIV with both SARS-CoV-2 infection and TB disease had an increased risk of death compared to patients with only SARS-CoV-2 infection (aHR 8.90, 95% CI 1.47-53.96, p=0.017), and to those with only TB disease (aHR 3.70, 95% CI 1.00-13.72, p=0.050).

**Table 4:** Mortality and adjusted risk of mortality at 6 months in people living with HIV presenting with symptoms of TB or of COVID-19 by diagnosis of TB disease and SARS-CoV-2 infection (N=475).

	n/N	%	aHR^†^	95% CI	p-value
**TB disease/SARS-CoV-2 infection**					
No TB disease and no SARS-CoV-2 infection	6/268	2.23	Ref		
Only TB disease	6/84	7.14	1.84	0.53–6.44	0.339
Only SARS-CoV-2 infection	2/103	1.94	0.77	0.15–3.93	0.750
Both SARS-CoV-2 infection and TB disease	6/32	18.75	6.83	1.75–26.72	0.006
**Sex**					
Women	5/299	1.67	Ref		
Men	15/188	7.98	2.98	0.97–9.16	0.057
**Age (per 1 year increase)**			1.02	0.98-1.06	0.284
**CD4 count** ^*^					
≥200 cells/µL	9/405	2.22	Ref		
<200 cells/µL	11/81	13.58	3.30	1.12–9.72	0.031
**BMI** ^*^					
<17	3/42	7.14	Ref		
≥17	13/438	2.97	0.73	0.18-2.91	0.654
**Diabetes** ^*^					
No	19/464	4.09	Ref		
Yes	1/12	8.33	3.56	0.38-33.16	0.264

† adjusted hazard ratio;

* Missing data for diabetes (n=11), BMI (n=7), CD4 count (n=1) were included in the model using specific categories (data not shown).

Schoenfield residuals test: p=0.480.

## Discussion

This prospective observational study found high proportions of positive SARS-CoV-2 IgG serology and SARS-CoV-2 infection among PLHIV with signs and symptoms of TB or advanced HIV in Uganda, Kenya, and South Africa. PLHIV with signs and symptoms of TB or of COVID-19, and with both SARS-CoV-2 infection and TB had higher mortality than PLHIV with only SARS-CoV-2 infection, only TB, or none of them. To date, there is limited evidence about the risk of mortality from triple co-infected patients and these results reveal or confirm several key findings with potential implications for clinical practice and public health policy.

Our findings revealed high positive SARS-CoV-2 IgG seroprevalence by the end of the study period (January-April 2022) and two years of pandemic, with substantial variations among the three countries studied. South Africa showed the highest seroprevalence (71%), while Uganda and Kenya rates were of 57% and 40%, respectively. These numbers reflect the epidemic situation in these countries. The first COVID-19 cases in Kenya, Uganda, and South Africa were confirmed in March 2020 [25–27]. Seroprevalences of SARS-CoV-2 in the general population have been reported to be 9.1% in Kenya [28], 15.6% in Uganda [29] and 27% in South Africa [30] by September, November and December 2020 respectively, after the first waves, and up to 70% in urban communities of South Africa, by November 2021, after the third wave [31]. A meta-analysis of standardized seroprevalence studies in Africa found that SARS-CoV-2 seroprevalence had increased from 3% in April–June 2020 to 65% in July–September 2021 [32]. More than half of participants who had an initial positive serology test and who repeated the test at the end of the study had a negative serology result at 6 months, which may indicate that the real proportion of people exposed to SARS-CoV-2 is higher than the proportion observed. Although test accuracy may vary with time since infection and severity of infection [33], and negative serologies may be due to early serology after symptoms onset, this finding along with the low proportion of vaccinated participants with initial negative SARS-CoV-2 and positive result after 6 months, may also indicate that a relevant proportion of PLHIV may not be protected from new SARS-CoV-2 infection, 6 months after previous infection or vaccination, and that viral transmission may persist.

Moreover, immunosuppression appeared to affect SARS-CoV-2 seropositivity rates. Patients with severe immunosuppression (<200 cells per μL) exhibited lower seropositivity rates than those with higher CD4 cell counts. This finding suggests that immunosuppressed individuals may have a weaker immune response to infection or vaccination, emphasizing the need for tailored vaccination strategies such as additional doses or more frequent vaccinations for this population [11,34–36].

Vaccination rates were very low in the studied population. Three quarters of the study visits (and serological testing) were done before August 2021 when vaccination against COVID-19 was very limited or not accessible to the general population in the three countries. Additionally, vaccination hesitancy may have also contributed [37].

SARS-CoV-2 infection and TB disease among PLHIV with symptoms of TB or COVID-19 was high, with 27.5% of the patients having SARS-CoV-2 infection (probable or confirmed), and 23.8% diagnosed with TB. This highlights the significant overlap of the clinical syndromes and symptoms among these two diseases and the difficulties to diagnose using only clinical symptoms. This underscores the importance of COVID-19 testing and TB investigations in individuals presenting with symptoms that can be attributed to any of these diseases.

PLHIV with symptoms of TB or of COVID-19, who were co-infected with both TB and SARS-CoV-2 exhibited higher mortality rates compared to those with SARS-CoV-2 infection, or only HIV. This increased risk mortality risk persisted after adjusting for age, sex, BMI, CD4 count, and country. However, COVID-19 vaccination rates were very low at the time of the study and mortality may be lower in populations with higher immunization coverage. These findings are consistent with previous research highlighting the increased mortality of patients with both TB and SARS-CoV-2 infection [4,5,8–13], underscoring the importance of active testing for both TB and SARS-CoV-2 in PLHIV [38] and comprehensive care for patients with concurrent HIV, TB, and SARS-CoV-2 infections [39].

Strengths of the study include multi-country study setting and the prospective design. The study population reflects the situation in the general ambulatory HIV population in the countries studied where the proportion of people living with HIV who were on ART in 2022 was 94% in Kenya, 84% in Uganda and 75% in South Africa [40]. A limitation of the study is that part of the individuals eligible for SARS-CoV-2 PCR test, declined the test, which resulted in loss of information and potential selection bias, though the characteristics of the participants who accepted and refused the test were similar. Seriously ill individuals were more represented among those tested, which may have potentially led to a higher overall mortality. PCR test refusal was mainly due to the strict restrictive measures applied in some countries for people diagnosed with COVID-19 (p.e. hospitalization). In addition, though PCR testing certainly missed some patients with SARS-CoV-2 infection, the definition of probable SARS-CoV-2 infection may have also classified as infected, patients without SARS-CoV-2 infection. Finally, the study was not powered to detect statistical differences by CD4 count or TB diagnosis. Lack of statistical association does not necessarily mean that the association does not exist.

In conclusion, these findings underscore the importance of targeted routine SARS-CoV-2 testing in PLHIV with symptoms of TB, and SARS-CoV-2 vaccination, especially for those with severe immunosuppression. PLHIV with COVID-19 and TB have an increased risk of mortality and would benefit from a comprehensive management approach and close monitoring. As there are increased reports of complications post-COVID-19, and SARS-CoV-2 has propensity to resurge, further research is needed to better characterize the triple infections, understand the dynamics of SARS-CoV-2 infection in HIV-positive individuals with TB, and to explore the potential benefits of tailored vaccination approaches.

## Supporting information

S1 Text Inclusivity in global research checklist. (DOCX)

S1 Fig Causal diagram to identify potential confounders of the association between mortality and SARS-CoV-2 infection and TB in people living with HIV. (TIF)

S1 Table Clinical-demographical characteristics at initial visit of participants who accepted and who refused SARS-CoV-2 PCR testing. (DOCX)

S2 Table Positivity of SARS-CoV-2 serology during the study period in people living with HIV. (DOCX)

S3 Table Description of the 20 deceased participants among people living with HIV with symptoms of TB or of COVID-19. (DOCX)
