# Supplementary material for: COVID-19, tuberculosis, and HIV triad: a prospective observational study in ambulatory patients in Kenya, Uganda, and South Africa
Source: PLOS Glob Public Health. 2025 Apr 23;5(4):e0004471. doi: 10.1371/journal.pgph.0004471 (PMC12017567; doi:10.1371/journal.pgph.0004471)
Supplement: S1 Text — (DOCX) [file pgph.0004471.s001.docx]

**S1 Text: Inclusivity in global research checklist**

**Ethical considerations, permits and authorship**

Provide details as to who granted permissions and/or consent for the study to take place in the Methods section of your manuscript. This should include the names of all ethics boards, governmental organizations, community leaders or other bodies that provided approval for the study. If individuals provided approval refer to these people by their role or title but do not list their name(s).

Reported on page number: 9

If there were any deviations from the study protocol after approval was obtained please provide details of these changes in the Methods section of your manuscript.

Reported on page number: No deviations

Did this study involve local collaborators that are residents of the country where the research was conducted or members of the community studied? If you do not have any authors from said communities, please provide an explanation for this below.

Answer: Yes, local collaborators are included.

**Human subjects research (e.g. health research, medical research, cross-cultural psychology)**

Did you obtain written informed consent from a representative of the local community or region before the research took place? How did you establish who speaks for the community? Details of written informed consent obtained from study participants should be reported separately in the Methods section of your manuscript.

Answer: Community Advisory Boards (CAB) and traditional village representatives were consulted before the research took place. Written informed consent was not obtained from a representative of the local community.

How did members of the local community provide input on the aims of the research investigation, its methodology, and its anticipated outcome(s)?

Answer: Meetings with CABs and traditional village representatives were held to explain the aims of the research study, the methods, and the expected outcome, and to give the opportunity to community representatives to provide input.

When engaging with the local community, how did you ensure that the informed consent documents and other materials could be understood by local stakeholders?

Answer: Informed consents and other ddocuments were translated into the local languages to ensure that they could be understood by local stakeholders.

Will the findings of the research be made available in an understandable format to stakeholders in the community where the study was conducted (e.g. via a presentation, summary report, copies of publications, etc.)? Please provide details of how this will be achieved.

Answer: Posters summarizing the study aims, methods and main results and written in local language were made available to the community.
