# Supplementary figures and images for: COVID-19, tuberculosis, and HIV triad: a prospective observational study in ambulatory patients in Kenya, Uganda, and South Africa
Source: PLOS Glob Public Health. 2025 Apr 23;5(4):e0004471. doi: 10.1371/journal.pgph.0004471 (PMC12017567; doi:10.1371/journal.pgph.0004471)

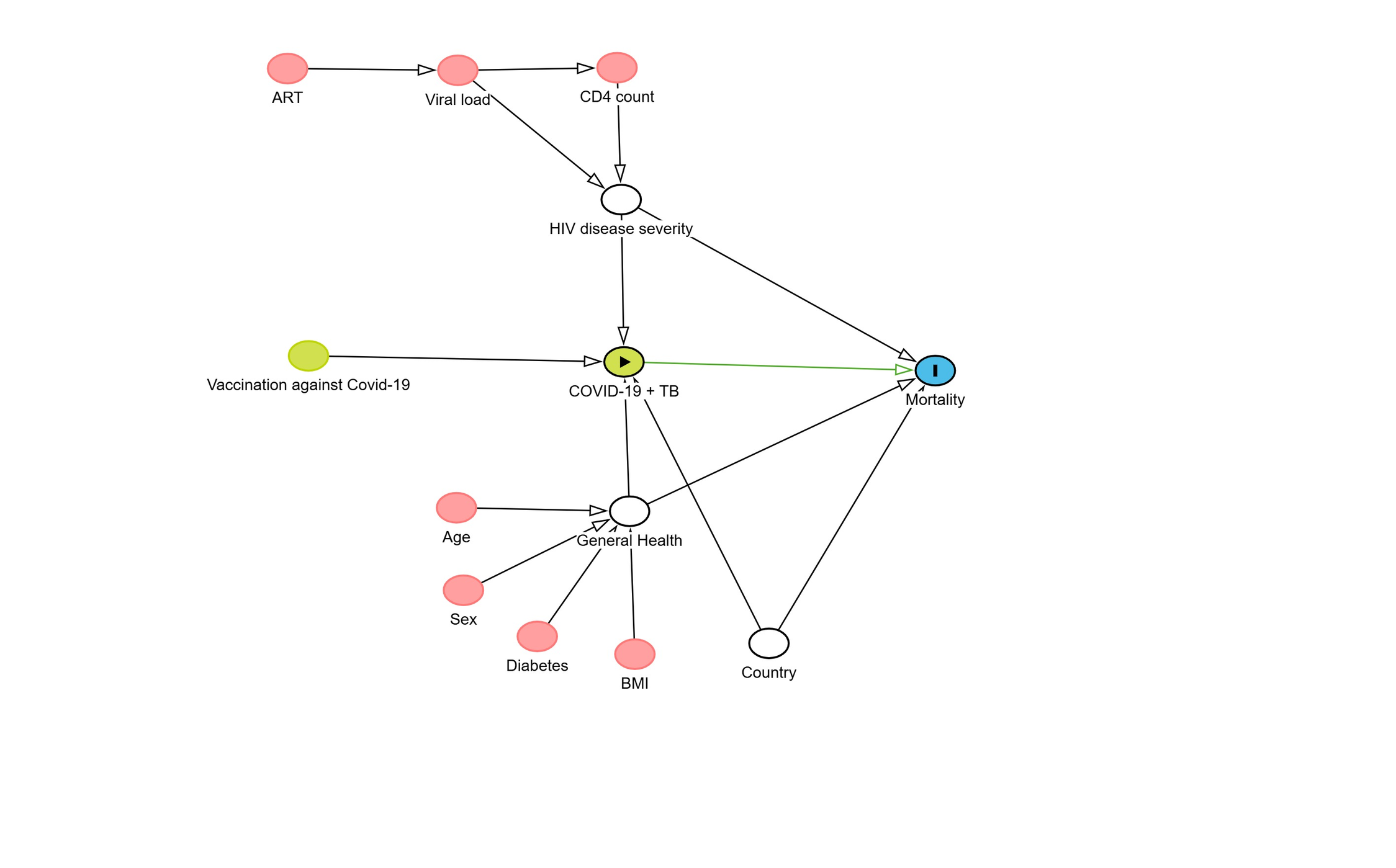

Supplement: S1 Fig — (TIF) [file pgph.0004471.s002.tif]
