# Supplementary material for: COVID-19, tuberculosis, and HIV triad: a prospective observational study in ambulatory patients in Kenya, Uganda, and South Africa
Source: PLOS Glob Public Health. 2025 Apr 23;5(4):e0004471. doi: 10.1371/journal.pgph.0004471 (PMC12017567; doi:10.1371/journal.pgph.0004471)
Supplement: S1 Table — (DOCX) [file pgph.0004471.s003.docx]

**S1 Table: Clinical-demographical characteristics at initial visit of participants who accepted and who refused SARS-CoV-2 PCR testing.**

|  | **Patients tested for SARS-CoV-2 PCR**  **(N=487)** | |  | **Patients not tested for SARS-CoV-2 PCR**  **(N=346)** |  |  |
| --- | --- | --- | --- | --- | --- | --- |
|  | n | % or IQR | | n | % or IQR | p-value |
| **Demographics** |  |  | |  |  |  |
| Women | 299 | 61.4 | | 192 | 55.5 | 0.088 |
| Men | 188 | 38.6 | | 154 | 44.5 |  |
| Age, median [IQR] | 42 | 34–52 | | 44 | 35–54 | 0.182 |
| **CD4 count** |  |  | |  |  |  |
| Median, cells/µL [IQR] | 555 | 313–784 | | 518.5 | 289–766 | 0.259 |
| <200 cells/µL | 81 | 16.6 | | 58 | 16.8 | 0.912 |
| ≥200 cells/µL | 405 | 83.2 | | 284 | 82.1 |  |
| Missing | 1 | 0.2 | | 4 | 1.2 |  |
| **Viral load** |  |  | |  |  |  |
| ≤1000 copies/mL | 327 | 67.1 | | 210 | 60.7 | 0.295 |
| >1000 copies/mL | 21 | 4.3 | | 19 | 5.5 |  |
| Missing | 139 | 28.5 | | 117 | 33.8 |  |
| **Antiretroviral therapy** |  |  | |  |  |  |
| Yes | 442 | 90.8 | | 305 | 88.2 | 0.149 |
| No | 39 | 8.0 | | 38 | 11.0 |  |
| Missing | 6 | 1.2 | | 3 | 0.9 |  |
| **Primary study group** |  |  | |  |  |  |
| Symptoms of TB | 483 | 99.2 | | 346 | 100 | 0.146 |
| No symptoms of TB, advanced HIV disease | 4 | 0.8 | | 0 | 0.0 |  |
| **Body-Mass Index** |  |  | |  |  |  |
| <17 kg/m² | 42 | 8.6 | | 32 | 9.2 | 0.193 |
| 17–18.4 kg/m² | 37 | 7.6 | | 39 | 11.3 |  |
| 18.5–24.9 kg/m² | 259 | 53.2 | | 180 | 52.0 |  |
| 25.0–29.9 kg/m² | 74 | 15.2 | | 60 | 17.3 |  |
| ≥30 kg/m² | 68 | 14.0 | | 35 | 10.1 |  |
| Missing | 7 | 1.4 | | 0 | 0.0 |  |
| **Clinical examination** |  |  | |  |  |  |
| Temperature ≥38°C | 16 | 3.3 | | 8 | 2.3 | 0.408 |
| Pulse >100 beats per min | 85 | 17.5 | | 52 | 15.0 | 0.352 |
| Respiratory rate >20 breaths per min | 148 | 30.4 | | 122 | 35.3 | 0.139 |
| Systolic blood pressure <90 mmHg | 11 | 2.3 | | 9 | 2.6 | 0.750 |
| Seriously ill | 34 | 7.0 | | 9 | 2.6 | 0.005 |
| **Country** |  |  | |  |  |  |
| Uganda | 273 | 56.1 | | 173 | 50.0 | <0.001 |
| Kenya | 79 | 16.2 | | 126 | 36.4 |  |
| South Africa | 135 | 27.7 | | 47 | 13.6 |  |
