# Supplementary material for: COVID-19, tuberculosis, and HIV triad: a prospective observational study in ambulatory patients in Kenya, Uganda, and South Africa
Source: PLOS Glob Public Health. 2025 Apr 23;5(4):e0004471. doi: 10.1371/journal.pgph.0004471 (PMC12017567; doi:10.1371/journal.pgph.0004471)
Supplement: S2 Table — (DOCX) [file pgph.0004471.s004.docx]

**S2 Table: Positivity of SARS-CoV-2 serology during the study period in people living with HIV.**

|  | Overall |  | Uganda |  | Kenya |  | South Africa |  |
| --- | --- | --- | --- | --- | --- | --- | --- | --- |
|  | n/N | % | n/N | % | n/N | % | n/N | % |
| September-December 2020 | 30/319 | 9.4 | 15/206 | 7.3 | 13/91 | 14.3 | 2/22 | 9.1 |
| January-April 2021 | 83/678 | 12.2 | 59/398 | 14.8 | 15/236 | 6.4 | 9/44 | 20.5 |
| May-August 2021 | 103/574 | 17.9 | 53/309 | 17.2 | 46/223 | 20.6 | 4/42 | 9.5 |
| September-December 2021 | 83/453 | 18.3 | 56/271 | 20.7 | 16/150 | 10.7 | 11/32 | 34.4 |
| January-April 2022 | 78/131 | 59.5 | 47/83 | 56.6 | 4/10 | 40.0 | 27/38 | 71.1 |
