# Supplementary material for: COVID-19, tuberculosis, and HIV triad: a prospective observational study in ambulatory patients in Kenya, Uganda, and South Africa
Source: PLOS Glob Public Health. 2025 Apr 23;5(4):e0004471. doi: 10.1371/journal.pgph.0004471 (PMC12017567; doi:10.1371/journal.pgph.0004471)
Supplement: S3 Table — (DOCX) [file pgph.0004471.s005.docx]

**S3 Table: Description of the 20 deceased participants among people living with HIV with symptoms of TB or of COVID-19.**

| **COVID-19 & TB diagnosis** | **SARS-CoV-2 infection** | **TB disease** | **Age range (years)** | **Sex** | **Hypertension** | **Diabetes** | **Seriously ill** | **BMI range (Kg/m2)** | **CD4 count (cells/µL)** | **Respiratory rate (/min)** | **Systolic blood pressure (mmHg)** | **Months to death** | **Reported possible cause of death** |
| --- | --- | --- | --- | --- | --- | --- | --- | --- | --- | --- | --- | --- | --- |
| Cov/TB | Confirmed | Confirmed | 35-44 | Male | No | No | Yes |  | <200 | >20 | <90 | 0.1 | HIV, TB, Covid-19 related complications |
| Cov/TB | Confirmed | Probable | 35-44 | Male | No | No | Yes |  | <200 | >20 | >=90 | 0.0 | Advanced HIV, TB, Covid-19 related complications |
| Cov/TB | Confirmed | Probable | 35-44 | Male | No | No | No | 18.5-24.9 | <200 | <=20 | <90 | 0.8 | Advanced HIV, TB |
| Cov/TB | Probable | Probable | 25-34 | Male | No | No | No | 18.5-24.9 | ≥200 | <=20 | >=90 | 1.5 | Blood transfusion reaction (reported by family) |
| Cov/TB | Probable | Probable | 25-34 | Male | No | No | No | 18.5-24.9 | <200 | <=20 | >=90 | 1.7 | HIV related illness |
| Cov/TB | Probable | Probable | 55-64 | Female | No | Yes | Yes |  | ≥200 | <=20 | >=90 | 0.2 | Kidney failure |
| Cov/No TB | Probable | No | 35-44 | Male | No | No | No | 18.5-24.9 | ≥200 | >20 | <90 | 1.6 | HIV related illness |
| Cov/No TB | Probable | No | 55-64 | Male | No | No | Yes | <17 | <200 | >20 | <90 | 0.9 | Advanced HIV |
| No Cov/TB | No | Confirmed | 25-34 | Male | No | No | No | 18.5-24.9 | <200 | <=20 | >=90 | 3.2 | Murdered |
| No Cov/TB | No | Confirmed | 35-44 | Male | No | No | Yes | 18.5-24.9 | <200 | >20 | >=90 | 0.3 | Advanced HIV, TB, anaemia |
| No Cov/TB | No | Confirmed | 55-64 | Male | No | No | No | 18.5-24.9 | ≥200 | <=20 | >=90 | 1.1 | Multi Drug Resistant TB |
| No Cov/TB | No | Probable | 55-64 | Female | No | No | No | 18.5-24.9 | <200 | <=20 | >=90 | 0.8 | Advanced HIV, TB, Cervical cancer |
| No Cov/TB | No | Probable | 44-54 | Male | No | No | No | <17 | ≥200 | >20 | >=90 | 0.1 | Pneumonia |
| No Cov/TB | No | Probable | 55-64 | Male | No | No | Yes |  | <200 | >20 | >=90 | 6.3 | Covid-19 related complications (reported by family) |
| No Cov/No TB | No | No | 55-64 | Male | No | No | No | 18.5-24.9 | ≥200 | >20 | >=90 | 2.6 | HIV related illness (reported by family) |
| No Cov/No TB | No | No | 25-34 | Male | No | No | No | 18.5-24.9 | <200 | <=20 | >=90 | 2.4 | Liver disease (reported by family) |
| No Cov/No TB | No | No | 45-54 | Male | No | No | No | 18.5-24.9 | <200 | >20 | >=90 | 4.4 | Pneumonia |
| No Cov/No TB | No | No | 45-54 | Female | No | No | No | <17 | ≥200 | >20 | >=90 | 3.9 | Throat cancer |
| No Cov/No TB | No | No | 55-64 | Female | No | No | No | 18.5-24.9 | ≥200 | >20 | >=90 | 1.3 | Low blood pressure and dehydration |
| No Cov/No TB | No | No | 35-44 | Female | No | No | No | 18.5-24.9 | ≥200 | >20 | >=90 | 5.9 | Drug overdose - suicide |
